# The neuropharmacology of sleep paralysis hallucinations: serotonin 2A activation and a novel therapeutic drug

**DOI:** 10.1007/s00213-018-5042-1

**Published:** 2018-10-05

**Authors:** Baland Jalal

**Affiliations:** Department of Psychiatry and Behavioural and Clinical Neuroscience Institute, University of Cambridge, Cambridge, UK

**Keywords:** Sleep paralysis, Hypnogogic, Hypnopompic, Hallucinations, Serotonin 2A, Hallucinogenic drugs, Mystical experiences, Ketanserin, Treatment

## Abstract

Sleep paralysis is a state of involuntary immobility occurring at sleep onset or offset, often accompanied by uncanny “ghost-like” hallucinations and extreme fear reactions. I provide here a neuropharmacological account for these hallucinatory experiences by evoking the role of the serotonin 2A receptor (5-HT_2A_R). Research has shown that 5-HT_2A_R activation can induce visual hallucinations, “mystical” subjective states, and out-of-body experiences (OBEs), and modulate fear circuits. Hallucinatory experiences triggered by serotonin—serotonergic (“pseudo”) hallucinations, induced by hallucinogenic drugs—tend to be “dream-like” with the experiencer having insight (“meta-awareness”) that he is hallucinating, unlike dopaminergic (“psychotic” and “life-like”) hallucinations where such insight is lost. Indeed, hallucinatory experiences during sleep paralysis have the classic features of serotonergic hallucinations, and are strikingly similar to perceptual and subjective states induced by hallucinogenic drugs (e.g., lysergic acid diethylamide [LSD] and psilocybin), i.e., they entail visual hallucinations, mystical experiences, OBEs, and extreme fear reactions. I propose a possible mechanism whereby serotonin could be functionally implicated in generating sleep paralysis hallucinations and fear reactions through 5-HT_2A_R activity. Moreover, I speculate on the role of 5-HT_2C_ receptors vis-à-vis anxiety and panic during sleep paralysis, and the orbitofrontal cortex—rich with 5-HT_2A_ receptors—in influencing visual pathways during sleep paralysis, and, in effect, hallucinations. Finally, I propose, for the first time, a drug to target sleep paralysis hallucinations and fear reactions, namely the selective 5-HT_2A_R inverse agonist, pimavanserin. This account implicates gene HTR2A on chromosome 13q as the underlying cause of sleep paralysis hallucinations and could be explored using positron emission tomography.

I have heard (but not believed), the spirits of the dead

May walk again. If such thing be, thy mother

Appeared to me last night; for ne’er was dream

So like a waking.

—Shakespeare

## Introduction

During rapid eye movement (REM) sleep, we experience vivid dreams. If we were to act out these dreams, we would risk hurting ourselves. So the brain has an ingenious solution: it leaves our body temporarily paralyzed. This paralysis is triggered by the pons (the pontine reticular formation) and ventromedial medulla that suppress skeletal muscle tone via interneurons of the spinal cord, through the inhibitory neurotransmitters GABA and glycine (Brooks and Peever 2012; Jalal and Hinton 2013; Kandel et al. 2000). However, the perceptual and motor aspects of REM sleep can occasionally decouple such that the sleeper begins to awaken before muscle paralysis has waned. The result is a curious condition called sleep paralysis, where the person is temporarily paralyzed (from REM postural atonia)—yet perceptually alert (or semi-alert) (Hobson 1995). Sleep paralysis victims often complain of hypnogogic and hypnopompic hallucinations, such as seeing space aliens and shadow-people in their bedroom (Cheyne et al. 1999a, b; Jalal et al. 2014b, 2015a, 2017, in press[a]; Jalal and Ramachandran 2014, 2017; McNally and Clancy 2005).

While the mechanism underlying sleep paralysis atonia is established (Brooks and Peever 2012), in comparison, relatively little is known about the pathophysiology of the uncanny hallucinations that can accompany these episodes. During sleep paralysis, for instance, there is a desynchrony between motor-execution (efference) and sensory input from the body (afference), resulting in massive deafferentation. This neural deafferentation may lead to “body image” distortions, entailing a functional disturbance of the multisensory processing of body and self at the temporoparietal junction (TPJ) and the right superior parietal lobule (SPL)—structures crucial for the construction of a neural representation of the body (Jalal and Ramachandran 2014). This account is broadly consistent with the finding that disrupting the TPJ using focal electrical stimulation can induce the feeling of an illusory “other” shadow-like person mimicking one’s body postures (Arzy et al. 2006), and that hyperactivity in the temporoparietal cortex of patients with schizophrenia can lead them to misattribute their own actions to others (Farrer et al. 2004). One hypothesis has also implicated the mirror neuron system, and the interaction between several brain regions, including the prefrontal cortex and sensory feedback, as contributing factors in triggering these hallucinations (Jalal and Ramachandran 2017). (On the mirror neuron system see also, Ramachandran 2012; Rizzolatti et al. 1996, 2001). It is indeed plausible that sleep paralysis hallucinations (“ghosts” and out-of-body experiences [OBEs]) arise from the interaction of multiple brain systems and the synergistic influence of several mechanisms.

To date, no account has been provided for the possible neuropharmacological basis of these “ghostly” hallucinations during sleep paralysis. As we shall see, serotonin 2A receptor (5-HT_2A_R) activation may play a crucial role in inducing these hallucinations.

## The role of orexin-hypocretin in promoting wakefulness

The neuropeptide orexin (orx-a/hcrt1 and orx-b/hcrt2)—originating in the hypothalamus—is central for keeping us awake. Orexin neurons turn on when we are awake and switch off when we sleep. A neurodegenerative loss of these “wakefulness neurons” results in the debilitating sleep disorder, narcolepsy (Bayer et al. 2004). It is hardly surprising that narcoleptic patients have difficulties staying awake. Orexin neurons in the hypothalamus project to various brain regions, including somatosensory, visual, motor, and cingulate cortices, where their excitatory effects promote wakefulness. They stimulate noradrenaline neurons in the locus coeruleus, histamine neurons, and cholinergic basal forebrain neurons (Brown et al. 2002; Eggermann et al. 2001; Eriksson et al. 2001; Hagan et al. 1999; Horvath et al. 1999; Huang et al. 2001; Ivanov and Aston-Jones 2000; see also Bayer et al. 2004; for a review, see Sakurai 2007). Orexin neurons also project to the dorsal raphé nucleus (DRN) to excite serotonin (5-HT) neurons (Aghajanian and Marek 1999). The DRN is virtually a “hub” for serotonin neurons (Liu et al. 2002). One feature of serotonin neurons in the DRN is that they stop firing during REM sleep, suggesting that the DRN functions as the REM sleep inhibitory area (Ursin 2002). In fact, the primary role of serotonin is to promote wakefulness and inhibit REM sleep.

## Serotonin 2A receptor activation: hallucinations, mystical experiences, OBEs, personal meaning, and paranoia

The serotonin receptors activated by serotonin—mediating both excitatory and inhibitory neurotransmission—include seven major subtypes, namely 5-HT_1–7_. Moreover, 5-HT_1_ receptors include 5-HT_1A_, 5-HT_1B_, and 5-HT_1D_; also, 5-HT_2_ receptors include 5-HT_2A_, 5-HT_2B_, and 5-HT_2C_ subtypes (e.g., Riva 2016; for a review, see Saulin et al. 2012).

Serotonin 2A (5-HT_2A_) receptors are of special interest. Molecular, pharmacological, and neuroimaging research demonstrate the central role of 5-HT_2A_ receptors in mediating visual processing and visual hallucinations. Indeed, there are a high number of 5-HT_2A_ receptors in the visual cortex (Gerstl et al. 2008; Moreau et al. 2010). Alterations of the density of 5-HT_2A_ receptors in the visual cortex of patients with schizophrenia and Parkinson’s disease are associated with experiencing visual hallucinations (González-Maeso et al. 2008; Huot et al. 2010). Pimavanserin, the 5-HT_2A_ receptor inverse agonist, is used to treat visual hallucinations in Parkinson’s disease (Meltzer et al. 2010). Interestingly, 5-HT_2A_ receptors mainly mediate the visual hallucinations—and indeed uncanny mystical experiences and altered states of consciousness—that are induced by hallucinogenic drugs like lysergic acid diethylamide (LSD), mescaline, and psilocybin (e.g., Griffiths et al. 2008; Vollenweider and Kometer 2010; on hallucinogens vis-à-vis 5-HT_2A_R, see also Nichols 2004). Research has shown that 5-HT_2A_ receptor activation induces visual hallucinations by increasing cortical excitability and altering visual-evoked cortical responses and that these effects of psilocybin on the visual system are blocked with the 5-HT_2A_ receptor antagonist ketanserin (Kometer et al. 2013).

5-HT_2A_R activation also selectively affects self-representation in fronto-parietal cortical regions, including the default mode network (DMN; Raichle et al. 2001) (e.g., Tagliazucchi et al. 2016; Carhart-Harris et al. 2012). The DMN is known to mediate processing of self-related information and neural self-constructs (Buckner et al. 2008). For instance, as noted above, the TPJ (a region of the DMN) is directly implicated in OBEs and hallucinating human-like figures (Arzy et al. 2006). The fact that 5-HT_2A_ receptors are distributed in higher-level cortical networks such as the DMN including the TPJ is consistent with findings that hallucinogenic drugs like psilocybin and LSD (5-HT_2A_R agonists) can induce OBE-type events, e.g., feelings of derealization and “ego dissolution” (e.g., Lebedev et al. 2015; Tagliazucchi et al. 2016). Also, one study in patients with depersonalization disorder found that short-term hallucinogen use could induce chronic symptoms of depersonalization (i.e., having a “fragmented sense of self”) (Simeon et al. 2003).

Moreover, research suggests that 5-HT_2A_ receptor activation underlies the propensity to ascribe meaning to “meaningless” stimuli. A recent study (Preller et al. 2017) found that activation of 5-HT_2A_ receptors, when induced by LSD—aside from generating hallucinations and feelings of “separateness from the self” (i.e., OBE-type states), leads to greater attribution of personal relevance to otherwise meaningless cues. This indicates that the 5-HT_2A_ receptor might play a role in the dysfunctional “personal relevance attribution” seen in psychiatric disorders such as schizophrenia; that is, all too often, patients with schizophrenia and related psychotic disorders find personal meaning in situations or places most healthy people interpret as meaningless (Preller et al. 2017). In fact, this may explain why the 5-HT_2A_ receptor is upregulated in untreated patients with schizophrenia (González-Maeso and Sealfon 2009), and indeed, many second-generation antipsychotic drugs precisely have an antagonistic effect on 5HT_2A_ receptors, stressing the possible role of 5HT_2A_ receptor activation in schizophrenia (Meltzer and Massey 2011; Rolland et al. 2014). Consistent with previous research, the aforementioned study found that the 5HT_2A_R antagonist ketanserin was able to block these effects of 5-HT_2A_ receptors (Preller et al. 2017).

Serotonin 2A receptors are also densely concentrated in the limbic system and are important for mediating the emotion of fear. There is evidence showing that the processing of emotionally salient information is modulated by the 5HT_2A_ receptor and that the expression of these receptors constitutes a trait related to anxiety (Frokjaer et al. 2008). Individuals with higher serotonin activity tend to have more fearful personalities, and interestingly, animals with a deficiency of 5-HT_2A_ receptors lack normal fear reactions (Nelson 2010). One study found that the 5-HT_2A_ receptor exerts influence on medial prefrontal regions which regulate the amygdala during fight-or-flight reactions (Fisher et al. 2009). Unsurprisingly, individuals under the influence of psilocybin may occasionally have extreme fear reactions and experience paranoia (Griffiths et al. 2006). The fear experienced when under the influence of hallucinogenic drugs manifests as hallucinations, paranoid delusions, and panic-like attacks. Taken together, the 5-HT_2A_ receptor appears to drive both visual hallucinations and the propensity to ascribe meaning to “meaningless” cues, and likewise profoundly modulates circuits intertwining fear and mystical experiences.

## Sleep paralysis hallucinations and serotonin 2A receptor activation

Ordinarily when waking from sleep, “arousal systems” become activated: orexin-producing neurons project both directly to cortical sites and critically the DRN (the so-called REM sleep inhibitory area) (Aghajanian and Marek 1999); in the DRN, these orexin neurons excite serotonin-producing neurons that in turn promote cortical arousal (Bayer et al. 2004; on serotonergic effects on REM sleep, see Pace-Schott 2008). By comparison, during sleep paralysis, there is likewise a transition from sleep (i.e., REM) to wakefulness, which involves activation of the same neural circuitry (i.e., wake-promoting neurons), but unlike normal wakefulness, the sleeper remains temporarily paralyzed (due to ongoing postural atonia), in spite of such activation of cortical and behavioral arousal mechanisms (i.e., wakefulness). That is, polysomnogram (PSG) recordings show that sleep paralysis is characterized by the curious persistence of REM atonia (indexed by a tonic electromyogram [EMG]) into wakefulness (i.e., shown by abundant bursts of “waking” alpha electroencephalogram [EEG] patterns) (Takeuchi et al. 1992). In other words, sleep paralysis is a unique dissociated state with simultaneous elements of both REM and wakefulness (different from other sleep states)—i.e., with the sleeper being “immobilized” yet perceptually awake (or “semi-awake”). It is plausible that during sleep paralysis, the activation of the serotonergic arousal system (i.e., phasic elevation of serotonin [i.e., via REM off cells]) to trigger perceptual wakefulness causes serotonin over-activity in the brain of the paralyzed sleeper. Serotonin could thus possibly be functionally implicated in generating sleep paralysis hallucinations through 5-HT_2A_ receptor activity. Over-activation of 5-HT_2A_ receptors during this peculiar “REM sleep-wake” state may in turn lead to massive increase in cortical excitability (e.g., in the visual cortex), elevated glutamate release in the neocortex affecting fronto-stratial glutamatergic pathways (including via medial prefrontal regions with high density of 5-HT_2A_ receptors, e.g., in layer V pyramidal cells; see Aghajanian and Marek 1999), and enhanced amygdala activation. (Research specifically suggests that 5-HT_2A_R activation may enhance glutamate release (i.e., unto layer V pyramidal neurons) via presynaptic activity as opposed to a postsynaptic mechanism [Aghajanian and Marek 1999; for related work, see Marek and Aghajanian 1998a, b].) Indeed, while serotonin as an endogenous neurotransmitter is not regarded as hallucinogenic per se, metabolites of serotonin have a high affinity for 5-HT_2A_ receptors and can trigger hallucinations in humans (Schmid and Bohn 2010). If this hypothesis is correct, and 5-HT_2A_R activation is functionally implicated in sleep paralysis hallucinations through the mechanism described here (or via a different mechanism), one should clearly see comparable neuropharmacological effects on the brain—and in turn subjective states of consciousness—both during sleep paralysis and when under the influence of hallucinogenic drugs. Below examples are provided of the similarities between subjective and perceptual states reported during sleep paralysis and those induced by drugs (5-HT_2A_ receptor agonists) such as LSD and psilocybin.

## Serotonergic hallucinations and sleep paralysis perceptual states

Serotonergic hallucinations (e.g., induced with psychedelic drugs) are sometimes referred to as “pseudo-hallucinations” or nonpsychotic hallucinations, and tend to be “dream-like” in nature. The experiencer can have perfect insight (“meta-awareness”) into the fact that these hallucinations are not real (Studerus et al. 2011; van der Zwaard and Polak 2001). On the contrary, hallucinations induced predominantly by D_2Rs_ activation (“dopaminergic hallucinations”) are experienced as more “life-like” and crisp—as indistinguishable from real life—and with the person having lost complete insight into the fact that the hallucinations are not real. Dopaminergic hallucinations are also known as “psychotic hallucinations” and commonly seen in schizophrenia (Gaebel and Zielasek 2009). It is worth adding here that hallucinations induced via distinct pathways, that is, either 5-HT_2A_, D_2Rs_, or primarily NMDA blockage (“glutaminergic hallucinations”), can have partial overlapping neurobiological mechanisms. As noted, the 5-HT_2A_ receptor may enhance glutamate release in prefrontal regions and LSD has been found to stimulate dopamine receptors, indicating the possible involvement of dopamine systems in LSD-induced hallucinations (Aghajanian and Marek 1999; Creese et al. 1975; see also, Vollenweider et al. 1998). But as described, the distinction in tonality between serotonergic “pseudo-hallucinations” and dopaminergic “psychotic hallucinations” is nonetheless phenomenologically robust (e.g., Aghajanian and Marek 1999; see also Rolland et al. 2014).

Hallucinatory experiences during sleep paralysis have the classic features of serotonergic hallucinations (“pseudo-hallucinations”) and are similar to those induced by hallucinogenic drugs. For example, sleep paralysis hallucinations tend to be “dream-like”—to the extent that the event itself sometimes is interpreted as a dream, especially so in cultures where there is no explanation or socio-cognitive framework for the experience (Fukuda et al. 2000). Moreover, during sleep paralysis, the sufferer can have ample awareness that he is in fact hallucinating (although this is not always the case) (i.e., “I know I am merely hallucinating this ‘ghost’ in front of me but it still feels real”) (e.g., Jalal et al. 2014b). Moreover, like hallucinogenic drugs, sleep paralysis is commonly associated with experiencing hallucinations that are “mystical” and “otherworldly” in nature. As noted, they often include seeing and sensing malevolent supernatural beings and having altered states of consciousness. Such OBEs can entail feelings of “dissociation” and “separation” from the “self” (e.g., autoscopy, derealization, and depersonalization states) (e.g., Cheyne and Girard 2009; Jalal and Ramachandran 2017). Like hallucinations produced by LSD for instance, hallucinatory experiences during sleep paralysis can include sensory distortions and the perception of colors and surfaces moving (e.g., Jalal et al. 2015a).

## Sleep paralyzes hallucinations and “personal relevance attribution”

As described elsewhere (e.g., Jalal 2016), during sleep paralysis, the person may experience a series of co-occurring symptoms such as chest pressure and pain, difficulty breathing, spasms in limbs, and seeing a human-like shadowy figure. This shadowy figure is somewhat analogous to a Rorschach inkblot in that it is ambiguous per se. But during sleep paralysis, it is possible that 5-HT_2A_ receptor activation could lead to the attribution of greater personal meaning to such endogenously generated cues (e.g., human-like shapes) and symptoms, for instance, drawing from autobiographical memories. This might be similar to the dysfunctional “personal relevance attribution” seen in psychotic disorders. In effect, these multisensory cues (visual, tactile, kinetic, etc.) are more likely to be interpreted in a personalized way and incorporated into an elaborate narrative, say, as a bedroom intruder (e.g., “Freddy Krueger”) approaching the helpless sleeper, pressing on his chest, and holding down his arms and legs, while attempting to rape or strangle him to death. Like experimentally induced multisensory experiences and illusions, for instance the rubber hand illusion (RHI), the Bayesian logic of all perceptual systems may play a role here (Botvinick and Cohen 1998; Jalal et al. 2015b), by allowing for top-down influence; the brain regards it as highly improbable that these somatic sensations and visual cues co-occur by random chance, and therefore builds a personally salient and meaningful narrative to account for them. Interestingly, given the link between serotonin and schizophrenia (González-Maeso and Sealfon 2009; Meltzer and Massey 2011; Rolland et al. 2014), multisensory illusions like the RHI are amplified in psychotic patients such as schizophrenics (Thakkar et al. 2011).

## Sleep paralyses: paranoia and panic-like fear reactions

As commonly seen with hallucinogenic drugs, sleep paralysis almost always produces an overwhelming sense of fear, and like LSD and psilocybin, manifesting as terrifying hallucinations, paranoid delusions, and panic-like reactions (Cheyne and Pennycook 2013; Jalal et al. in press[b]; Jalal and Hinton 2013; Solomonova et al. 2008). Indeed, the experiencing of fear during sleep paralysis is one of its key features—reported in both the presence and absence of hallucinations (e.g., Jalal and Hinton 2013). There is no evidence to suggest that extreme fear reactions during sleep paralysis diminish after multiple episodes or having prior knowledge about the neurological roots of the experience—sleep paralysis on its own appears to activate amygdaloid fear circuitry at a core level. As such, the fear is not merely a result of realizing that one is paralyzed, catastrophic cognitions about the event (e.g., “I am dying”), and unpleasant somatic symptoms or hallucinations, although these almost certainly contribute to the underlying fear and can create a positive feedback loop (see “panic-hallucination” model; Jalal 2016, 2017).

The elevated fear levels seen during sleep paralysis are consistent with the well-established link between 5-HT_2A_ receptor activation and fear reactions as described above (e.g., Fisher et al. 2009; Nelson 2010). It might explain why sleep paralysis almost always is reported as being terrifying worldwide, and rarely as a neutral or benign experience. Whether one experiences (serotonin-induced) fear and paranoia when taking hallucinogenic drugs depends on one’s environment and emotional state at the time of drug administration (Katz et al. 1968). These factors may play a role during sleep paralysis as well. But as sleep paralysis includes inherently unpleasant features (paralysis, chest pain, and difficulty breathing, potentially leading to activation of “threat hypervigilance systems” due to feelings of helplessness, etc. (Cheyne 2001; Jalal 2016), fear might be the most likely outcome. If one’s culture attributes sleep paralysis to pernicious supernatural forces (e.g., a “demonic attack”), such underlying fear could be enhanced due to further amygdaloid serotonin release. That is, research has found that stress leads to increased serotonin release in the amygdala of rats (Kawahara et al. 1993). Consistent with this, research has shown that sleep paralysis sufferers in Egypt who interpret their experience as a supernatural attack report greater fear during the event than Danish sufferers, who for the most part interpret sleep paralysis as merely a physiological oddity (Jalal and Hinton 2013; Jalal et al. 2014b).

## Serotonin subsystems and anxiety and panic during sleep paralysis: 5-HT_2C_R activation

Undoubtedly, identifying the behavioral function of specific 5-HT subsystems in humans will be an important step towards illuminating the exact role of serotonin vis-à-vis sleep paralysis and fear-panic reactions. One interesting model of serotonin function, for instance, highlights the paradoxical role of 5-HT_2C_ receptors in anxiety and panic, i.e., in response to distal versus more proximal (or imminent) threats, mediated by brain-aversion systems including the amygdala, hypothalamus, and the periaqueductal gray matter (PAG) (Deakin and Graeff 1991; see also Deakin 2013). According to this view, serotonin acts as an “anticipatory anxiety system” (or “don’t panic yet” system) where distal threats generate anxiety via the amygdala and frontal cortex, while restraining premature activation of (PAG) fight-flight reactions via serotonin (5-HT_2C_ receptors) in the DRN. On the other hand, when “threats” are proximal (e.g., believing that death is imminent or when detecting touch, pain, or suffocation sensations [hypoxia and hypercapnia]—all of which are common during sleep paralysis [e.g., Jalal 2016; Cheyne et al. 1999b]), serotonin activates the PAG resulting in panic reactions. This account of serotonin functioning was nicely illustrated in a functional magnetic resonance imaging (fMRI) study by Mobbs et al. (2007), in which healthy volunteers played a video game where they had to control a stimulus through a visual maze, while avoiding annihilation by a threatening “ghosts” stimulus. Consistent with Deakin and Graeff’s (1991) model, the authors found that when the “ghost” was at a distance, the amygdala and frontal cortex were activated, whereas more imminent threats by the “ghost” activated PAG fight-flight mechanisms. I propose that in addition to the possible role of 5-HT_2A_ receptors in mediating sleep paralysis fear-anxiety reactions (e.g., Frokjaer et al. 2008), these functional principals of 5-HT_2C_R activation might drive anxiety (via the amygdala and DRN) and panic reactions (via the PAG) during sleep paralysis. Indeed, analogous to Mobbs et al.’s (2007) video game, sleep paralysis hallucinations often include scenarios where “ghosts/demons” manifest as distal threats (e.g., lurking in the bedroom corner) and/or as proximal/imminent threats (e.g., “ghost attacks” resulting in choking and suffocation sensations, pain and spasm in limbs, etc.).

## The orbitofrontal cortex and sleep paralysis visual hallucinations

It is worth noting that until recently it was thought that one’s emotional state is totally independent of the brain’s visual system. However, it is now believed that affective states such as fear are sources of top-down penetration in that they can directly influence our perceptual system (Barrett and Bar 2009; O’Callaghan et al. 2017). There are several pathways whereby this penetration could occur, including the magnocellular system and projections to the orbitofrontal cortex (Pessoa and Adolphs 2010). Neuroimaging evidence points to the orbitofrontal cortex in particular as a region that can generate top-down predictions about the emotional value of visual stimuli (Shenhav et al. 2013). The orbitofrontal cortex is a densely connected association site that receives input from various sensory modalities including limbic centers; it is strategically located to integrate cross-modal information pertinent to the interpretation of visual stimuli (Rolls 2004). The amygdala rich with 5-HT_2A_ receptors is connected to the orbitofrontal cortex, and may provide an important source of input to the orbitofrontal cortex about the affective value of stimuli (Kawahara et al. 1993; Pessoa and Adolphs 2010; Roy et al. 2012; see also O’Callaghan et al. 2017). It is of particular interest that 5-HT_2A_ receptors are densely concentrated in the orbitofrontal cortex (see Robbins et al. 2006). It is plausible that during sleep paralysis, the 5-HT_2A_R**-**induced amygdaloid fear circuitry mediated by the orbitofrontal cortex directly influences visual pathways and consequently hallucinations (i.e., by creating a fearful scenario that matches the individual’s emotional state). This activation of the brain’s “threat-hyper vigilance system” could facilitate top-down influence, for example, by allowing for endogenously generated somatic sensations and ambiguous cues to be interpreted as threatening and exogenously driven—i.e., as a petrifying “ghostly bedroom intruder.”

## The HTR2A gene and sleep paralysis hallucinations

It is possible that individuals with increased 5-HT_2A_ receptor density (e.g., in certain brain regions) are more prone to hallucinating during sleep paralysis. These hallucinations might be associated with the expression of the HTR2A gene on chromosome 13 that produces higher receptor density. The gene coding for HTR2A is located on the long arm of chromosome 13 (i.e., 13q) (Williams et al. 1996; see also Chen et al. 1992). Consistent with the role of serotonin in triggering hallucinations, whole-genome linkage scans have found an association between schizophrenia and markers on chromosome 13q (for a meta-analysis, see Badner and Gershon 2002). Also, alterations in the HTR2A gene and expression of it may be vulnerability factors predisposing to mood disorders, such as anxiety and depression (e.g., Islam et al. 2004). Research in particular has investigated abnormalities of 5-HT signaling vis-à-vis anxiety (e.g., Abrams et al. 2005; Keck et al. 2005; see also Unschuld et al. 2007). The fact that the 5-HT_2A_ receptor has been implicated in psychopathology, including anxiety disorders, is of interest (Millan 2003). That is, anxiety is associated with hallucinating during sleep paralysis: One study among Egyptian college students found that having visual hallucinations during sleep paralysis was associated with trait anxiety symptomatology (Jalal and Hinton 2015). Moreover, Hinton et al. (2005) found that highly traumatized Cambodians have extremely high rates of hallucinations during sleep paralysis (i.e., 91% of episodes) compared to general populations (e.g., Jalal and Hinton 2013).

## Conclusions

It is important to add that while sleep paralysis causes much fear and anxiety in general, the vast majority of episodes are benign and unrelated to serious pathology. Thus, there is often no need for treatment except in cases of repetitive and fearful episodes. Although clinical trials are lacking, selective serotonin reuptake inhibitors (SSRIs) are one of the most commonly used drugs in clinical practice to treat distressing sleep paralysis (Sharpless 2016; see also Sharpless and Doghramji 2015). This is unsurprising given serotonergic modulation of the sleep/wake cycle and, crucially, the anxiolytic effects of SSRIs.

In light of this hypothesis, I propose for the first time a drug to selectively target sleep paralysis hallucinations and fear reactions, namely pimavanserin. Pimavanserin is a highly selective 5-HT_2A_ receptor inverse agonist; it has by comparison approx. 40-fold lower affinity for the 5-HT_2C_ receptor. This makes it a promising drug candidate for targeting sleep paralysis hallucinations. As noted, pimavanserin has been found to attenuate l-DOPA-induced hallucinations (and delusions) in Parkinson’s disease. (Another candidate drug is the 5-HT_2A_ receptor antagonist ketanserin. It is possible that ketanserin blocks the hallucinatory features of sleep paralysis, as it does hallucinogenic drugs like psilocybin [Kometer et al. 2013].) This proposed neuropharmacological account implicates gene HTR2A on chromosome 13q as the underlying cause of sleep paralysis hallucinations and could be explored using positron emission tomography.
